# H_2_S mediates increased interleukin (IL)-1β and IL-18 production in leukocytes from patients with periodontitis

**DOI:** 10.1080/20002297.2019.1617015

**Published:** 2019-05-20

**Authors:** Amina Basic, Giovanni Serino, Åsa Leonhardt, Gunnar Dahlén

**Affiliations:** a Oral Microbiology and Immunology, The Sahlgrenska Academy, University of Gothenburg, Gothenburg, Sweden; b Department of Periodontology, Södra Älvsborgs Hospital, Borås, Sweden

**Keywords:** Periodontal disease, H_2_S, IL-1β, IL-18, PBMCs, bismuth plaque test, qPCR

## Abstract

**Background**: The mechanisms involved in the interplay between the bacteria and the host cells in periodontitis are not fully understood.

**Aim**: To investigate the effect of the bacterial metabolite H_2_S on the pro-inflammatory cytokines interleukin (IL)-1β and IL-18 from periodontitis patients and healthy controls, and to evaluate the composition of the subgingival microbiota with its capacity to produce H_2_S.

**Methods**: Subgingival bacterial samples from patients with periodontitis (N=32) and healthy controls (N=32) were investigated for H_2_S production and bacterial composition. Peripheral blood mononuclear cells (PBMCs) were cultured in the presence/absence of 1mM H_2_S for 24h and cytokine concentrations were measured.

**Results**: Subgingival plaque from periodontitis patients had more H_2_S producing bacteria and produced more H_2_S, than healthy controls. PBMCs exposed to H_2_S secreted significantly more IL-1ß and IL-18 (*p*<0.0001) than untreated control PBMCs from both groups. PBMCs from the periodontitis patients secreted higher levels of the cytokines, both spontaneously (IL-1ß *p*=0.0001; IL-18 *p*=0.09) and after exposure to H_2_S (IL-1ß *p*=0.03; IL-18 *p*=0.04), which is a new finding not previously reported.

**Conclusions**: H_2_S, from the subgingival microbiota, can contribute to a host inflammatory response through secretion of the pro-inflammatory cytokines IL-1β and IL-18. Since this response differs between individuals, it may also reflect the susceptibility of the host to develop periodontitis.

Although the growth and activity of the subgingival microbiota are essential for periodontitis to develop, the specific role that the bacteria play in the inflammatory host response, which leads to periodontal destruction, remains unclear. The outcome of the interactions that occur between bacteria (and products/components thereof) and host cells is highly dependent upon the individual [1,2]. Host factors, such as heredity (both genetic and epigenetic) factors and lifestyle habits (e.g. smoking), are assigned high importance in the development and degree of the severity of periodontitis.

Previous studies have primarily focused on the presence and composition of the subgingival microbiota, including anaerobic and proteolytic bacterial species. Also, the lipopolysaccharides (LPS) of Gram-negative bacteria have been investigated as a major actor in the microbe-host interplay [3]. Less attention has been focused on the metabolites of bacteria, such as H_2_S. H_2_S is one end product of the proteolytic bacterial activity of the subgingival microbiota [4] that uses the proteins and peptides, including the amino acid cysteine, of the gingival crevicular fluid (GCF). H_2_S is produced by the degradation of cysteine by enzymes [5,6] of many bacterial species that are associated with periodontitis, such as *Porphyromonas gingivalis, Treponema denticola* and *Fusobacterium* spp [7]. Since higher levels of H_2_S have been detected at diseased sites, H_2_S has been suggested to serve as a marker for the proteolytic activity of the biofilm and also to be involved in the pathogenesis of periodontal disease [8,9]. H_2_S is toxic already at concentrations in the μM range [10] and has been shown *in vitro* to be able to decrease myeloperoxidase activity in polymorphonuclear leukocytes [11]. A recent study implies H_2_S to function as a bacterial defense mechanism by showing how the presence of high levels of bacterial H_2_S increases the resistance to immune-mediated killing [12].

An intensified and non-resolving inflammatory host response to subgingival microbiota is believed to cause tissue destruction in patients with periodontitis but the mechanisms that trigger and maintain this inflammation are not fully understood. Bacterial metabolites may contribute to this elevated response in susceptible individuals through increased secretion of cytokines, leading to aggravated periodontal destruction.

We hypothesize that H_2_S takes part in the pathogenesis of periodontitis by stimulating the secretion of pro-inflammatory cytokines from monocytes. In a previous *in vitro* study [13], we showed elevated secretion of interleukin (IL)-1β and IL-18 from human peripheral blood mononuclear cells (PBMCs) exposed to a hydrogen sulfide generator. The oral status of the donors was unknown. Large inter-individual variations in the cytokine responses were found, leading us to suggest that periodontitis patients secrete more IL-1β and IL-18 when exposed to bacterial H_2_S compared to healthy individuals, which may indicate a higher susceptibility to developing periodontitis.

The primary aim of the present study was to investigate the secretion of IL-1β and IL-18 from PBMCs of periodontitis patients and healthy controls after exposure of the cells to H_2_S. A second aim was to measure the capacity of subgingival bacteria to degrade cysteine and produce H_2_S, and to test the association between this H_2_S production and inflammation in the gingiva. Our data demonstrate that the bacterial samples from periodontitis patients have a higher capacity to produce H_2_S and that this production is correlated to the inflammation of the gingiva. In addition, the PBMCs of periodontitis patients secrete higher levels of pro-inflammatory cytokines both spontaneously and when exposed to H_2_S compared to healthy controls. This has not previously been reported.

## Materials and methods

### Subjects

This study was conducted in accordance with ethical principles of the World Medical Association Declaration of Helsinki and approved by the Regional Ethical Review Board (Dnr 871–15) in Gothenburg, Sweden. The periodontitis patients were recruited at the Department of Periodontology, Södra Älvsborgs Hospital, Borås, Sweden. All patients who were referred to the specialist clinic and who fulfilled the inclusion criteria were asked to participate. The subjects included in the healthy group were recruited at the Institute of Odontology, University of Gothenburg, Gothenburg, Sweden. The majority of the subjects in the healthy group were staff members at the dental clinics or research personnel. All participants were informed of the nature of the study both verbally and in writing. The subjects that agreed to participate in the study also signed informed consent forms.

The inclusion criteria for both groups were:

- Aged 40 years or older
- No use of antibiotics 3 months prior to the examination

The group-specific inclusion criteria were:

### Healthy controls

- At least 24 remaining teeth
- No history of periodontitis
- No Probing Pocket Depth (PPD) > 4 mm that could not be explained by other reasons than periodontitis, e.g. a partially erupted third molar, a crack in a root canal-treated tooth or a subgingival crown-dentin junction

### Periodontitis patients

- At least 14 remaining teeth
- Diagnosed with periodontitis (Bleeding on Probing (BoP) and attachment loss)
- PPD > 4 mm at least at 30% of the teeth

Three subjects in the healthy group and seven in the periodontitis group were taking medication for high blood pressure. Of these seven subjects, two had also diabetes. In total, three subjects in the periodontitis group had diabetes, but only one person in the healthy group. One subject in the periodontitis group and two in the healthy group had a cardiovascular disorder. In the periodontitis group, two patients reported that they suffered from arthritis. Five subjects in the periodontitis and healthy groups, respectively, used snuff, whereas smokers (N = 9) were only found in the periodontitis group.

### Clinical examination

The clinical examinations were performed by author GS on the periodontitis patients and by AB on the subjects who were defined as healthy. Patients were interviewed regarding their health and their use of tobacco (smoking and snuff), and medications. The registered data related to the number of remaining teeth, PPD > 4 mm at four sites per tooth, BoP, and Plaque Index (PI).

Seven of the subjects in the healthy control group had a gingival PPD of >4 mm, although each instance of tissue breakdown could be attributed to reasons other than periodontitis, e.g. a partially erupted third molar, a crack in a root canal-treated tooth or a subgingival crown-dentin junction.

### The bismuth test (BT)

The bismuth test (BT) was used to determine the ability of the microbiota to degrade cysteine and form H_2_S. One bacterial sample in each quadrant was taken from one of the deepest pockets in the periodontitis patients and from the mesial part of tooth 16, 21, 36, and 41 in the healthy group. The BT was performed as previously described [9]. Briefly, a mini-sponge (3M ESPE, Forsbergs Dental, Gothenburg, Sweden) was inserted in the subgingival pocket to collect the sample and moved to a well of a microtiter plate (96 MicroWell Plates Nunc, Roskilde Denmark) containing 100 μl of the bismuth solution (0.4 mM triethanolamine – HCl pH 8.0 (Fisher Scientific GTF AB, Gothenburg, Sweden)), 20 μM pyridoxal 5-phosphate monohydrate (VWR, Stockholm Sweden), 20 mM EDTA (Sigma-Aldrich Sweden AB, Stockholm, Sweden), 10 mM bismuth(III)chloride (Fisher Scientific), and 40 mM L-cysteine (Sigma-Aldrich). After 2 h of incubation at room temperature, a photograph was taken of the well. The color of the mini-sponge was registered by three individuals and scored as: no color change (0), small color change (1), medium color change (2), and high color change to a black sponge (3). The median score for the three observers was used.

### Bacterial sampling

Bacterial samples were taken from the same four sites as for the BT using two sterile paper points per sampling site for qPCR, and they were inserted into Eppendorf tubes and stored at – 20°C until the analyses. Aliquots of 200 μl 10 mM Tris buffer (pH 8.0) were added to the tubes, and the tubes were boiled for 10 min. The paper points were removed and the samples were stored at – 20°C until further analysis.

### Quantitative PCR (qPCR)

The samples were screened for five bacterial species associated with periodontitis. The amplification and detection were performed using the MiniOpticon^TM^ Real-Time PCR System with an MJ Mini^TM^ gradient thermal cycler (Bio-Rad Laboratories AB, Solna, Sweden). Data were processed and analyzed using the Bio-Rad CFX Manager. In a reaction mixture of 10 μl of Sso Fast^TM^ EvaGreen® Supermix (Bio-Rad) and 5 μl (1 μM) of each primer, 5 μl of the sample was added. The reactions were run in duplicate. The following primers (Invitrogen, Lidingö, Sweden) were used (forward and reverse, respectively): *Fusobacterium* spp., 5´-GGATTTATTGGGCGTAAAGC-3´ and 5´-GGCATTCCTACAAATATCTACGAA-3; for *P. gingivalis*, 5´-TGTAGATGACTGATGGTGAAAACC-3´ and 5´-ACGTCATCCCCACCTTCCTC-3´; for *Prevotella intermedia*, 5´-TTTGTTGGGGAGTAAAGCGGG-3´ and 5´-TCAACATCTCTGTATCCTGCGT-3´; for *Tannerella forsythia*, 5´-GCGTATGTAACCTGCCCGCA-3´ and 5´-TGCTTCAGTGTCAGTTATACCT-3´; and for *T. denticola*, 5´-TAATACCGAATGTGCTCATTTACAT-3´ and 5´-TCAAAGAAGCATTCCCTCTTCTTCTTA-3´ [14,15]. The reaction was run according to the protocol of Kuboniwa et al. [14]. The mean CT values were compared to standard curves to calculate the absolute quantifications of the samples.

### Blood samples

Peripheral blood samples were collected at the examination in both groups, before initiation of treatment for the periodontitis patients. The blood samples were analyzed for C-reactive protein (CRP), and transferrin at the Clinical Chemistry Laboratory of Sahlgrenska University Hospital, Gothenburg, Sweden. PBMCs were isolated and exposed to a hydrogen sulfide generator as previously described [13]. Briefly, PBMCs were isolated by centrifugation with Ficoll-Paque^TM^ Plus (GE Healthcare Bio-Sciences AB, Uppsala, Sweden), washed with PBS, and resuspended in Dulbecco’s Modified Eagle Medium plus GlutaMAX^TM^ (Gibco, Life Technologies, Paisley, UK) supplemented with 5% human serum (Sigma-Aldrich) and penicillin-streptomycin (Sigma-Aldrich). Aliquots that contained 2 × 10^6^ cells were cultured in 96-well microtiter plates in a humidified atmosphere with 5% CO_2_ at 37°C. The cells were exposed to 1 mM sodium hydrosulfide (NaHS; Fisher Scientific), for 24 h and compared with unexposed control cells. The culture supernatants were collected and stored at −80°C until all the samples were collected.

### Bio-Plex assay

Culture supernatants were analyzed for IL-1β and IL-18 with a customized panel that uses Luminex magnetic beads for the quantification and that allows both cytokines to be quantified simultaneously (Bio-Plex Pro^TM^ Human Cytokine Assay; Bio-Rad Laboratories, Hemel Hempstead, UK). Briefly, color-coded beads conjugated with capture antibodies were incubated with the samples, and a biotinylated detection antibody was added followed by the addition of streptavidin that was conjugated to the fluorescent indicator phycoerythrin. The bead color and mean fluorescence intensity was compared against a standard curve using the BioPlex 200 instrument with BioManager analysis software to determine the cytokine concentrations.

### Statistics

The initial *in vitro* experiments of a small pilot study with PBMCs derived from buffy coats [13] showed large variations in the levels of secreted IL-1β, from 0 μg/ml to 1,181 μg/ml. Based on these results, a power analysis was conducted. Since the periodontal status of the subjects who donated the buffy coat cells was unknown the calculations were based on the assumption that there is a difference between periodontitis patients and healthy controls.

The statistical analyses were achieved using the GraphPad Prism ver.7.0 software (GraphPad Inc., La Jolla, CA) and the IBM SPSS Statistics Software ver. 21 (SPSS Inc., Chicago, IL). In the tables, an independent sample *t*-test or Chi-square test for independence (Fisher’s exact test for subjects with PPD) was applied. The median was used for the results from the BT or the scores were assigned to low (score 0 and 1) and high (score 2 and 3) H_2_S production. A Chi-square test was used to explore the relationship between the BT and the periodontitis/healthy group. For the IL-1β and IL-18 results, the non-parametric Mann–Whitney test and the Wilcoxon signed-rank test were used. A *p*-value < 0.05 was considered statistically significant; *p* < 0.05*, *p* < 0.01**, *p* < 0.001***, *p* < 0.0001****.

## Results

### Clinical measurements

Sixty-four subjects were examined. One of the periodontitis patients chose not to donate a blood sample and was therefore excluded from the blood analyses. The characteristics of the subjects included in the study are presented in Table 1. In the periodontitis group, the mean PPD of the sampled sites was 6.4 ± 1.2 mm. All the sites sampled in the healthy group had a PPD of ≤4 mm. There was a clear clinical distinction regarding the inflammatory state of the gingiva between patients with periodontitis and healthy controls.

**Table 1.** Characteristics of the periodontitis group and the healthy control group.

Variable		Periodontitis (*N* = 32)	Healthy (*N* = 32)	*p*-Value
Women (%)		11 (34)	16 (50)	0.311
Age, mean ± SD (years)		54.3 ± 8.6	57.5 ± 8.6	0.146
Tobacco smoking (%)		9 (28)	0 (0)	0.002
Snuff use (%)		5 (16)	5 (16)	1
Remaining teeth, mean ± SD		24.9 ± 3.7	27.5 ± 0.9	< 0.001
PI,^1^ mean ± SD		40.5 ± 25.6	29.9 ± 18.3	0.063
BoP,^2^ mean ± SD		48.8 ± 22.0	19.1 ± 12.6	< 0.001
Subjects with PPD (%)^3^	>4 < 7 mm	3 (9)	6 (19)	< 0.001
	≥7 mm	29 (91)	1 (3)	

^1^Plaque Index ^2^Bleeding on Probing ^3^At least one pocket with Probing Pocket Depth (PPD) >4 < 7 mm or ≥7 mm

### Microbiology

**Figure 1.**
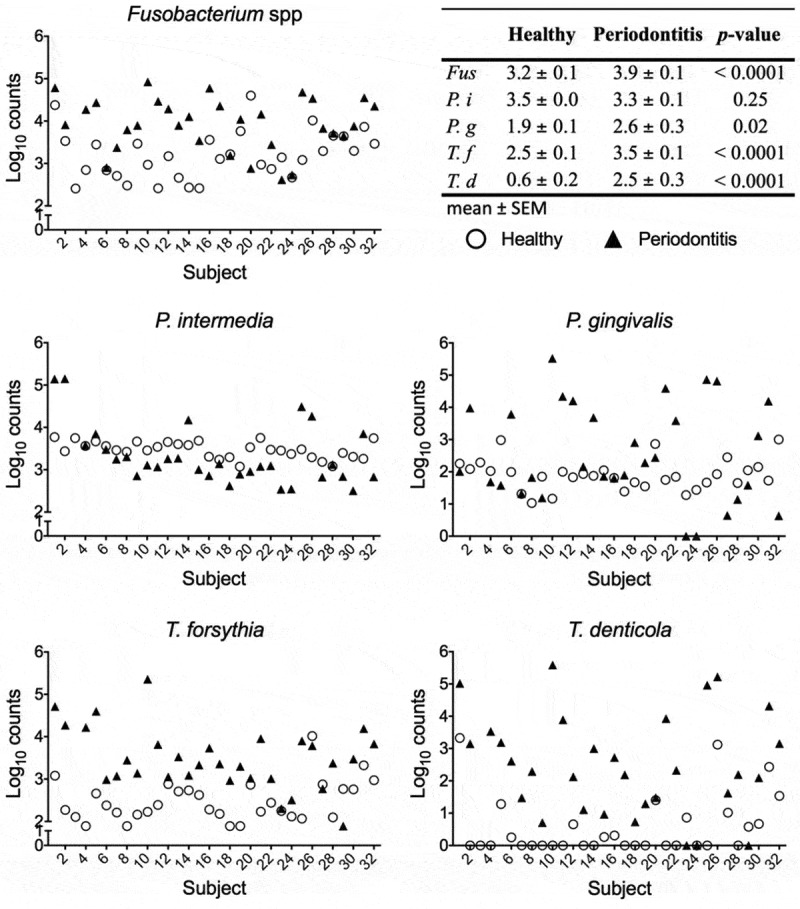
The presence of bacteria associated with periodontitis. The log_10_ counts for the bacterial species tested for each subject are shown (O Healthy, ▲ Periodontitis). The table shows a statistically significant difference between the two groups for all species except *P. intermedia*.

To verify that the sampled sites in periodontitis patients harbored a diseased microbiota with higher amounts of periodontitis-associated bacterial species than healthy sites, five bacterial species, *P. gingivalis, T. denticola, T. forsythia, Fusobacterium* sp., and *P. intermedia*, were quantified with qPCR. The tested bacterial species were detected in samples from subjects in both groups. Comparing the periodontitis group with the healthy group, there was a statistically significant difference in the number of bacteria for four out of the five bacterial species tested (Figure 1). *P. intermedia* was found in almost equal numbers in both groups. Thus, the bacterial numbers and composition of samples from the periodontitis patients were periodontitis-associated and distinct from samples in the healthy control group.

### H_2_S production

**Figure 2.**
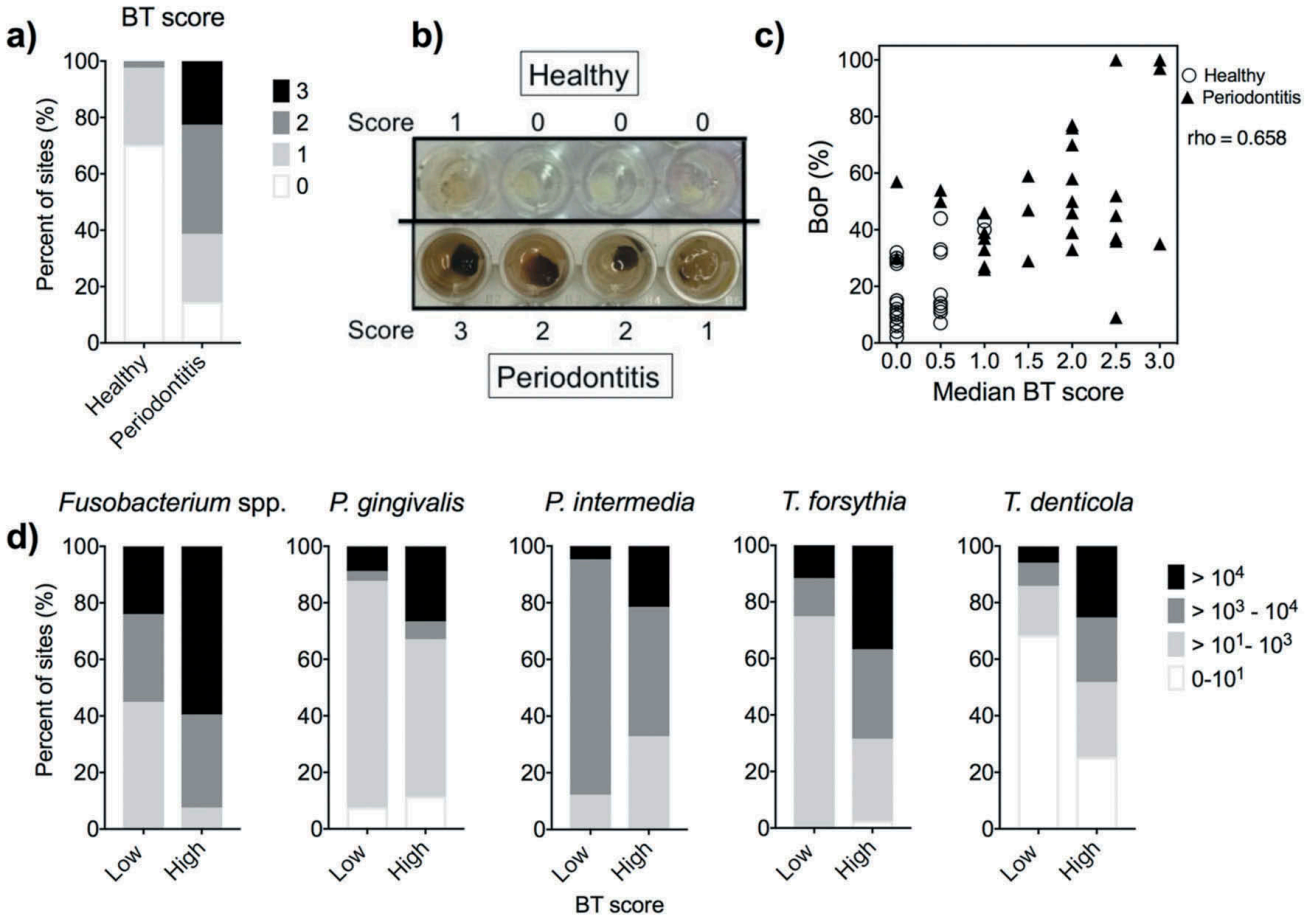
The results from the bismuth test (BT). (a) The bismuth test score of all sites is illustrated in the bars for healthy and periodontitis groups. The median of four sites/subject was 0 (range 0–1) for the healthy group and 2 (range 0–3) for the periodontitis group. (b) The photo is showing the BT of a subject in the healthy group and one from the periodontitis group. (c) BoP and BT scores (median) were tested positive for the correlation (rho = 0.658, p < 0.0001) (O Healthy, ▲ Periodontitis). (d) The BT score (high/low) is shown in relation to the bacterial counts for the five bacterial species tested.

To determine the capacity of the bacterial samples to produce H_2_S, the Bismuth test (BT) was used [9]. A representative photograph of the BT for samples from a periodontitis patient and a healthy control is shown in Figure 2(b). The group median of the individual median (four sites/subject) BT score was lower in the healthy group = 0 (range, 0–1) than in the periodontitis group = 2 (range 0–3) (*p* < 0.001, Chi-square test, Cramer’s V = 0.80) (Figure 2(a)). The relationship between clinical gingival inflammation, registered as BoP, and H_2_S production, registered as the median BT score for each individual, showed a strong positive correlation between these variables; rho = 0.658, N = 64, *p* < 0.0001 (Figure 2(c)). By comparing the BT score (high/low) to the log_10_ counts of bacteria (Figure 2(d)), a higher bacterial count was found at the sites in which the BT scores were high for all bacteria tested, with the exception of *P. intermedia*. Clearly, samples from periodontitis patients had a higher metabolic capacity to degrade cysteine and produce H_2_S. This capacity was correlated to the amount of clinical gingival inflammation.

### Cytokine production ± H_2_S

**Figure 3.**
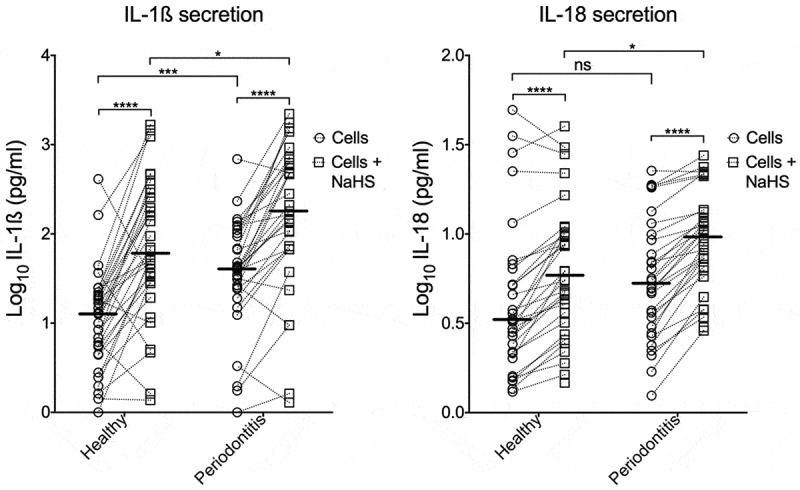
Cytokine secretion of PBMCs. The secretion of IL-1β and IL-18 from both unexposed PBMCs and exposed PBMCs to H_2_S. The PBMCs were sampled from patients with periodontitis and from healthy controls. The median is shown as a vertical line (IL-1β: 1.10 and 1.78 for healthy sites, 1.61 and 2.25 for periodontitis sites. IL-18: 0.52 and 0.77 for the healthy, 0.72 and 0.98 for periodontitis sites).

The secretion of the pro-inflammatory cytokines IL-1β and IL-18 from PBMCs was measured from both unexposed cells (i.e. spontaneous release) and cells exposed to the H_2_S generator NaHS for 24 h. The exposed cells showed statistically higher (*p* < 0.0001) levels of secretion of both IL-1β and IL-18 compared to unexposed control cells (Figure 3).

Unexposed cells from periodontitis patients secreted higher levels of IL-1β compared to the healthy controls (*p* = 0.0001). There was, however, no significant difference for spontaneous IL-18 secretion (*p* = 0.09). Also, the cells exposed to H_2_S secreted higher levels of IL-1β and IL-18 in the periodontitis group compared to the healthy group (*p*= 0.03 and *p*= 0.04, respectively).

The difference (delta of exposed vs unexposed) between the healthy and periodontitis group was not statistically significant for IL-1β (*p* = 0.18). A difference was however seen for IL-18 (*p* = 0.02).

To conclude, exposure to H_2_S resulted in higher levels of the pro-inflammatory cytokines, and the levels were higher from cells of periodontitis patients compared to cells from healthy controls.

### The impact of nicotine

Cigarette smokers were only found among the periodontitis patients, while snuff users were equally distributed in the two groups. Therefore, a test to exclude the nicotine users from the analyses of the cytokine secretion was conducted (Supplemental Figure S1). The results obtained were similar, although not always reaching statistical significance due to the underpowered nature after the exclusion of subjects. Thus, the results showed the same trend when nicotine users were excluded which suggests that nicotine use is of inferior importance in the regulation of IL-1β and IL-18 secretion from PBMCs *in vitro*.

## Discussion

The main outcome of this investigation is that the PBMCs of periodontitis patients secreted higher levels of IL-1β, both spontaneously and when exposed to H_2_S, as compared to healthy controls. Additionally, similar results were seen for IL-18 when exposed to H_2_S. To our knowledge, this has not previously been reported. In addition, the subgingival bacteria sampled from patients with periodontitis had a higher capacity to degrade cysteine and produce H_2_S, and comprised more periodontitis-associated species than the corresponding samples from the healthy controls. Exposure of PBMCs to H_2_S at a concentration expected to be present in diseased deep periodontal pockets [8] increased the levels of secreted L-1β and IL-18 in both groups. A correlation was seen between the BT scores and BoP. Taken together, these results suggest a pro-inflammatory role for bacterial H_2_S in the pathogenesis of periodontitis. Strong activity of the subgingival microbiota (which results in high levels of H_2_S and other metabolites of the bacterial activity, as well as LPS and enzymes) may contribute to an inflammatory response through increased secretion of IL-1β and IL-18. This response may be stronger in susceptible individuals, thereby enhancing periodontal destruction. Although this investigation shows that H_2_S exposure enhances the secretion of the cytokines *in vitro*, we suggest that this process occurs also *in vivo*, acting as one of the several mechanisms to enhance the inflammatory response through the activation of PBMCs and macrophages in the tissues. However, many other substances, such as various metabolites, enzymes, and LPS [16] exert pro-inflammatory activities that have similar effects on PBMCs [17], and it is the net effect of all these complex interactions that most likely leads to periodontitis [18].

In the present study, there were statistically significant differences in the secretion by unexposed cells of IL-1β between the two groups. The levels of IL-18 secretion showed the same trend but were not statistically significant for unexposed cells. When the cells were exposed to H_2_S, the secretion of IL-1β and IL-18 was increased from the cells of the majority of the subjects in both groups. These results are in accordance with those of our previous *in vitro* study of unknown blood donors [13] and confirm that H_2_S induces PBMCs to secrete pro-inflammatory cytokines. The level of secretion was, however, highly variable between the individuals tested, and some individuals (N = 9, IL-1β and N = 4, IL-18) actually secreted lower levels of IL-1β and IL-18 after H_2_S exposure than unexposed control cells. While the reason for this is unknown, it may indicate an immunosuppressive or toxic role for H_2_S in certain individuals. The concentration of applied H_2_S ought to determine the response of the PBMCs. It is tempting to propose that higher concentrations of H_2_S affect the PBMCs more than lower concentrations; although there is a maximal concentration before the cells undergo apoptosis or necrosis. While this needs to be investigated further, we have confirmed that the PBMCs remained viable after 24 h of exposure to 1 mM of the H_2_S generator NaHS [13].

Secretion of IL-1β and IL-18 from PBMCs is the result of two signals. The priming signal leads to the intracellular production of pro-IL-1β and pro-IL-18. The secondary signal activates a complex called the NLRP3 inflammasome and promotes the extracellular secretion of IL-1β and IL-18. We have previously shown that H_2_S contributes to elevated levels of the cytokines, although the results revealed lower levels compared to pre-exposure of the cells to LPS [13]. It is possible that the PBMCs from patients with periodontitis already had been primed and activated the primary signal, prior to sample collection, resulting in the higher secretion rates seen in our study. Similar priming has previously been documented in peripheral monocytes from periodontitis patients [19]. If the primed inflammatory response to H_2_S reported on cells from patients with periodontitis in this study is inherent, or acquired as a consequence of the chronic inflammatory response present around the teeth is not known. We found no differences (*p*= 0.224) in CRP between patients with periodontitis (mean 2.0 ± 2.5 mg/L) and healthy controls (mean 1.3 ± 1.9). CRP is an acute-phase protein that is produced during inflammation and reflects the inflammatory state of the individual. Similarly, low transferrin levels may indicate systemic inflammation, but no differences (*p*= 0.120) were seen in our study (mean 2.6 ± 0.3 g/L for patients with periodontitis compared to 2.5 ± 0.4 g/L for healthy controls). Altogether, these results suggest that there was no systemic inflammation present, and that the elevated response of the PBMCs may be an intrinsic feature.

While periodontal breakdown usually occurs differently between sites in periodontitis patients, a recent study [20] has indicated that there are no major differences in the local environment between sites with severe destruction as compared to a healthy site when investigating the cytokine profile. Zekeridou et al. [20] reported similar cytokine concentrations in the GCF for healthy and diseased sites in the same patient but noted a difference between periodontitis patients and healthy controls. The authors [20] proposed an individual inflammatory state in periodontitis patients that is not limited to diseased sites. These results support the general opinion that certain individuals are more prone to a stronger inflammatory response, which contributes to the breakdown of the supporting tissues around the teeth. The same study [20] reported no statistically significant differences in the IL-1β levels in the GCF between healthy subjects and subjects with periodontitis or in subjects with periodontitis before and after treatment. These results may be explained by the previously suggested ‘burst of activity theory’ where destruction of the supporting tissues occurs only during short periods of time in between periods of remission where bacteria and the host response are in balance [21]. During these bursts, the activity of the bacteria is high, with a high production of metabolites including H_2_S, and thus, therefore, the inflammatory host response is intensified.

qPCR was used to verify that the periodontitis patients harbored higher numbers of bacteria associated with periodontal disease. Although the periodontitis-associated species were found also in the healthy subjects, higher counts were noted for all except *P. intermedia* in the periodontitis patients. *P. intermedia* has previously been shown not to have as high H_2_S producing capacity as *Fusobacterium* spp [7,22]. The bacterial findings in the present study are similar to those reported by Kuboniwa et al. [14], except that we sampled more subjects and sites, and found additional bacteria-positive sites for *P. gingivalis* in healthy subjects, although the opposite result was obtained for *T. denticola*. This discrepancy may be due to the fact that the previous study [14] used a different qPCR technique with TaqMan probes. Furthermore, large inter-individual variations were seen in our study between subjects in both groups (Figure 1). Interestingly, the species that discriminated most clearly the two groups was the spirochete *T. denticola*, which was found in <50% of the healthy subjects but in the majority of the subjects in the periodontitis group. *T. denticola* is a known strong producer of H_2_S [7,22,23].

We were, however, primarily interested in the proteolytic activity of the oral microbes, rather than their compositions and numbers. Therefore, we used the BT (Figure 2), which proved to be able to discriminate between periodontitis patients and healthy controls. By investigating disease severity within the same patient, Morita and Wang [24] reported an association between the sulfide levels and the severity of the disease. H_2_S in deep periodontal pockets was reported by Persson [8], who detected H_2_S in all samples from pockets with PPD > 3 mm. However, the results of another study [25] failed to discriminate between shallow and deep periodontal pockets in relation to H_2_S. Furthermore, in our previous study [9], we found no association between the sulfide levels and PPD at the sampled site. Torresyap et al. [26] reported higher counts of the red-complex bacteria *P. gingivalis, T. denticola* and *T. forsythia* in sulfide-positive sites, which is consistent with our findings (Figure 2(d)). The somewhat higher bacterial counts reported in that study [26] could be due to the use of curettes (rather than paper points) for bacterial sampling. Interestingly, the same study [26] reported that shallow sulfide-positive sites harbored higher counts of red- and orange-complex species, indicating that the amount of sulfide may estimate the counts of these bacteria. Furthermore, Torresyap et al. [26] reported increases in the clinical parameters, including BoP, in sulfide-positive sites. A significant association between BoP and sulfide is also seen in our study (Figure 2(c)). However, one should keep in mind that the BoP reflects the percentage of all sites, while the BT score is a median of four sites. Taken together, sulfide levels are associated with bacterial counts and composition in addition to activity and inflammation, and not necessarily periodontitis and PPD.

Since smoking is associated with periodontitis, we tested to exclude the nicotine users from the analyses of the cytokine secretion and obtained similar results (Supplemental Figure S1). This indicates that nicotine does not play an essential role in the regulation of IL-1β and IL-18 secretion from PBMCs. A previous study [27] reported higher sulfide levels in the subgingival pockets of smokers, despite having similar bacterial counts to the non-smokers. In the present study, when categorizing the subjects of the periodontitis group into smokers (N = 9), snuff-users (N = 5) and non-smokers (N = 18), the median BT scores are 2.0, 2.5, and 2.0, respectively. Therefore, there was no apparent difference between smokers and non-smokers in BT scores.

In summary, the subgingival microbiota can contribute to inflammatory/immune responses by stimulating the host cells to secrete pro-inflammatory cytokines, such as IL-1ß and IL-18, upon exposure to H_2_S produced by the colonizing bacteria. The findings of this study have a potential clinical relevance in the detection of periodontitis susceptible individuals, but also disease active sites by the usage of BT as an indicator of bacterial proteolytic activity.
