# Supplementary material for: H2S mediates increased interleukin (IL)-1β and IL-18 production in leukocytes from patients with periodontitis
Source: J Oral Microbiol. 2019 May 20;11(1):1617015. doi: 10.1080/20002297.2019.1617015 (PMC6534246; doi:10.1080/20002297.2019.1617015)

**Supplemental Figure S1.** **Cytokine secretion of PBMCs from non-nicotine users**

The nicotine users, nine smokers in the periodontitis group and five snuff users in both groups, were excluded and the same analyses were performed as in Figure 3. Similarly, the cells that were exposed to H_2_S secreted more cytokines (p = 0.0002 for IL-1β in both groups and p = 0.0007 for IL-18 in the healthy group, p < 0.0001 in the periodontitis group). The unexposed cells secreted less IL-1β in the healthy group (p = 0.0055) but not statistically different concentrations of IL-18 (p = 0.1890). There were no statistical differences between the exposed cells in the two groups for IL-1β (p = 0.1080) and IL-18 (p = 0.1123). The vertical lines show the median of the group (IL-1β: 1.10 and 1.71 for healthy sites, 1.64 and 2.15 for periodontitis sites. IL-18: 0.56 and 0.79 for the healthy, 0.74 and 1.01 for periodontitis sites).


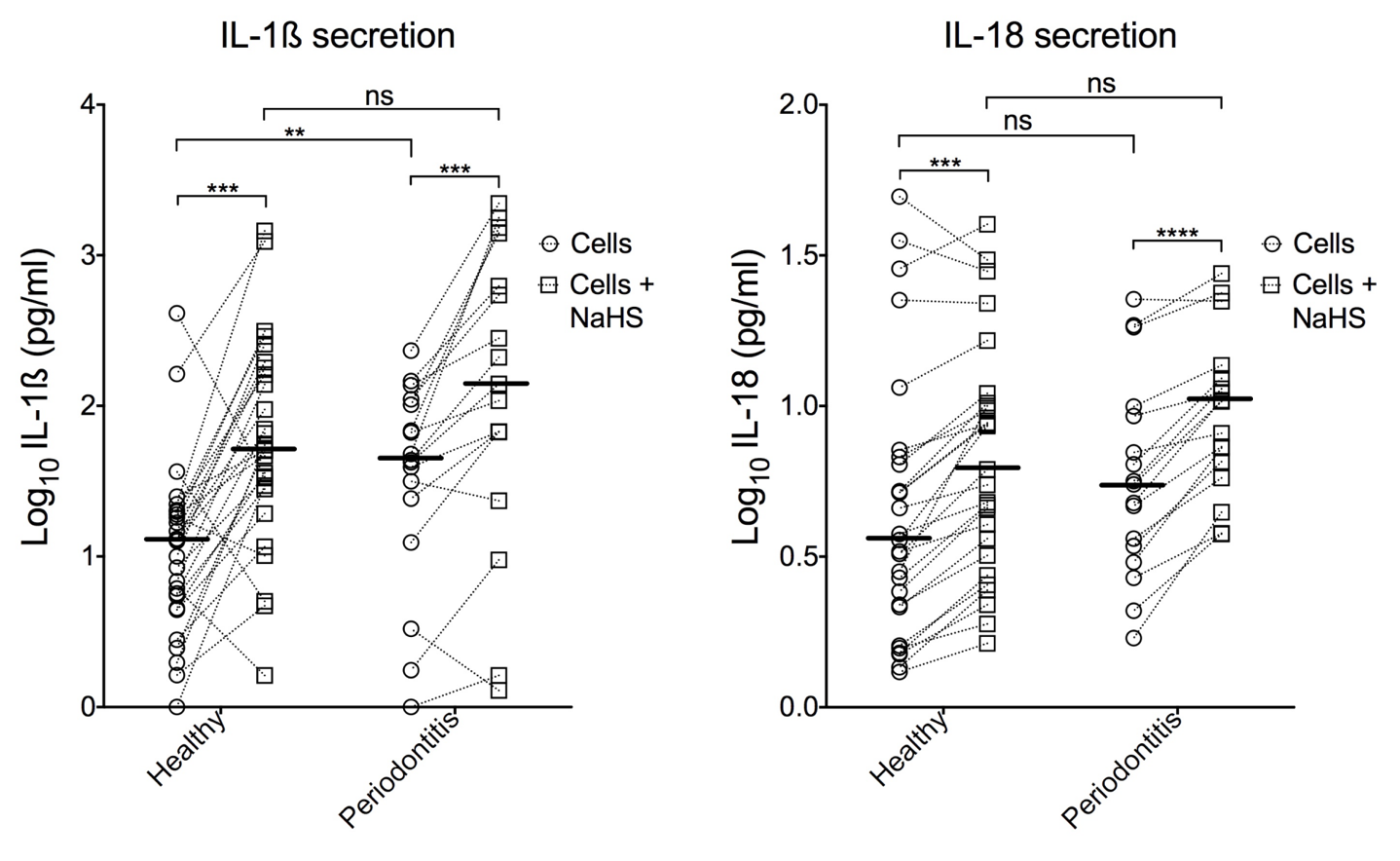

Supplement: Supplemental Material [file ZJOM_A_1617015_SM2744.docx]
